# Interactions between PDA-associated polymorphisms and genetic ancestry alter ductus arteriosus gene expression

**DOI:** 10.1038/s41390-021-01506-6

**Published:** 2021-04-09

**Authors:** Ronald I. Clyman, Nancy K. Hills, John M. Dagle, Jeffrey C. Murray, Keegan Kelsey

**Affiliations:** 1 Department of Pediatrics and Cardiovascular Research Institute, University of California San Francisco, San Francisco, CA USA; 2 Departments of Epidemiology and Biostatistics, and Neurology, University of California San Francisco, San Francisco, CA USA; 3 Department of Pediatrics, University of Iowa, Iowa City, IA USA

## Abstract

**Background:**

DNA polymorphisms in *PTGIS* and *TFAP2B* have been identified as risk factors for patent ductus arteriosus (PDA) in a population composed of preterm infants with European genetic ancestry but not in more genetically diverse populations.

**Goal:**

To determine if the effects of TFAP2B and *PTGIS* polymorphisms on ductus arteriosus (DA) gene expression differ based on genetic ancestry.

**Methods:**

DA from 273 human second trimester fetuses were genotyped for *TFAP2B* and *PTGIS* polymorphisms and for polymorphisms distributing along genetic ancestry lines. RT-PCR was used to measure the RNA expression of 49 candidate genes involved with DA closure.

**Results:**

Seventeen percent of the DA analyzed were of European ancestry. In multivariable regression analyses we found consistent associations between four PDA-related *TFAP2B* polymorphisms (rs2817399(A), rs987237(G), rs760900(C), and rs2817416(C)) and expression of the following genes: *EPAS1*, *CACNB2*, *ECE1*, *KCNA2*, *ATP2A3*, *EDNRA*, *EDNRB*, *BMP9*, and *BMP10*, and between the PTGIS haplotype rs493694(G)/rs693649(A) and *PTGIS* and *NOS3*. These changes only occurred in DA with European ancestry. No consistent positive or negative associations were found among DA samples unless an interaction between the polymorphisms and genetic ancestry was taken into account.

**Conclusion:**

*PTGIS* and *TFAP2B* polymorphisms were associated with consistent changes in DA gene expression when present in fetuses with European ancestry.

**Impact:**

- DNA polymorphisms in PTGIS and TFAP2B have been identified as risk factors for patent ductus arteriosus (PDA) in a population composed primarily of preterm infants with European genetic ancestry but not in more genetically diverse populations.
- The same PTGIS and TFAP2B polymorphisms are associated with changes in ductus gene expression when present in ductus from fetuses with European genetic ancestry.
- No consistent associations with gene expression can be found unless an interaction between the polymorphisms and genetic ancestry is taken into account.

## Introduction

In contrast with full-term infants, those born before 28 weeks’ gestation frequently fail to close their ductus arteriosus (DA) after birth. Persistent DA patency alters cerebral, mesenteric, and renal blood flow, impairs pulmonary mechanics, increases the risk of pulmonary hemorrhage, and prolongs the need for mechanical ventilation. Prior studies have shown that immature gestation, absence of antenatal glucocorticoid exposure, and mother’s self-identified race are the most consistent independent risk factors for identifying preterm newborn infants who fail to close their patent ductus arteriosus (PDA) either spontaneously or with inhibitors of prostaglandin production like indomethacin and ibuprofen.^1–5^ Both immature gestation and absence of antenatal betamethasone decrease the expression of a wide range of DA genes involved in oxygen-induced constriction (e.g., calcium channels, potassium channels, and endothelin signaling), contractile protein maturation, prostaglandin- and nitric oxide-mediated relaxation, and tissue inflammation and remodeling.^5–7^

There is growing evidence from monozygotic twin studies that genetic risk factors may act in concert with gestational age to alter the ability of the DA to close in preterm infants.^8,9^ We previously identified several single-nucleotide polymorphisms (SNPs) in genes encoding transcription factor AP-2 beta (*TFAP2B*, the gene mutated in Char syndrome) and prostacyclin synthase (*PTGIS)*, which are associated with isolated (non-syndromic) PDAs in preterm infants.^10^ *PTGIS* and its vasodilatory lipid product, prostacyclin (PGI2), play an important role in maintaining preterm DA patency.^11^ Similarly, TFAP2B, a transcription factor that regulates endothelin, hypoxia inducible factor 2-alpha (HIF2 alpha), and calponin, plays an important role in DA smooth muscle development.^10,12,13^ We previously examined one of the *TFAP2B* polymorphisms (SNP *rs2817399(A)*) that has been associated with persistent DA patency, for its effects on human fetal DA gene expression and found that it decreased several of the same calcium- and potassium-channel genes previously shown to be involved with oxygen-induced constriction of the DA.^6^

In contrast with our findings, two subsequent epidemiologic studies^14,15^ failed to find an association between the same SNPs we identified in *TFAP2B* and *PTGIS* and alterations in DA closure. Although differences in both the definition of PDA and the strategies used to treat the PDA might account for the discordant results between studies, another explanation might be the significant differences in genetic ancestry among the study populations. Ninety percent of mothers in our original Iowa-based, single center study self-identified as White/European ancestry.^10^ In contrast, 50 and 0% of the populations in the subsequent two studies self-identified as European ancestry.^14,15^ In addition, the Iowa study utilized a family-based approach, which is less susceptible to the effects of population stratification compared to the case–control design used in the latter studies.

We designed the following study to determine whether the PDA-associated SNPs in *TFAP2B* and *PTGIS* that we previously identified are indeed associated with unique alterations in gene expression. Our goal was to test the reproducibility of our prior findings in fetal DA obtained from a population with diverse genetic ancestry and to expand the list of genes that might be affected by the *TFAP2B* and *PTGIS* polymorphisms. We hypothesized that an interaction exists between the fetus’s genetic ancestry and the SNPs in *TFAP2B* and *PTGIS* such that the effects of the SNPs on gene expression only occur in DA with European genetic ancestry.

## Methods

We used de-identified DNA and RNA samples, collected as part of a prior study,^7^ to determine whether common genetic variants in *TFAP2B* and *PTGIS*, which have been associated with a PDA in preterm newborns, are associated with unique patterns of gene expression in the human fetal DA. The study was reviewed by the Institutional Review Board of the University of California San Francisco and given an exempt status.

### Tissue

Human tissue was obtained under the oversight of the Institutional Review Board at University of California San Francisco. Mid-gestation (13^0/7^–23^6/7^ weeks) human fetal DA and ascending aorta were collected from elective pregnancy terminations in healthy women with no known fetal abnormalities. Consent for the use of fetal tissue for research purposes was obtained by the clinic staff, who had been trained in human subjects’ protections. The consent for the use of fetal tissue for research purposes is separate from the consent for the clinical procedure. Researchers have no patient contact and only receive de-identified tissues.

Prostaglandins were not used during the terminations. Cervical ripening was performed with laminaria (compressed seaweed). Fetal tissue was immediately submerged in calcium- and magnesium-free phosphate-buffered saline at 4 °C following delivery. The DA and aorta were dissected in the chilled buffer solution and the isolated DA and aorta were snap frozen in liquid nitrogen (between 1.5 and 2 h after delivery). Gestational age was determined by fetal foot length.^16^ De-identified tissues were individually labeled and stored for later analysis. Individual samples were analyzed in “batches” of 90 samples. There was no “pooling” or combining of tissues during the analyses.

During the period of the study, women who donated tissue self-identified their racial origins to the clinic staff as White/European ancestry = 21%, Non-White/Non-European ancestry = 76%, and unknown = 3%. The data on self-reported racial origins were available solely as a population-level statistic. Individual descriptors were not linked to de-identified tissues samples. No clinical information was available for analysis.

### Preparation of total RNA, reverse transcription, and quantitative PCR

We examined the RNA expression of 49 “DA closure genes” in each of the 273 human DA samples (Table 1). The “DA closure genes” were chosen because: (1) their expression in the DA has previously been shown to differ from their expression in the aorta, and (2) their mutations or polymorphisms (or their pharmacologic inhibition) has been shown to affect DA closure (see refs. ^7^^,^^6^ for references for “DA closure genes”).

**Table 1:** Multivariable regression models examining the independent effects of gestational age and non-European genetic ancestry on the RNA expression of “ductus closure genes” (*n* = 273).

Genes/aliases	Regression coefficients for Gestation^a^							Regression coefficients for non-European ancestry^b^						
	*TFAP2B*	*TFAP2B*	*TFAP2B*	*TFAP2B*	*TFAP2B*	*TFAP2B*	*PTGIS*	*TFAP2B*	*TFAP2B*	*TFAP2B*	*TFAP2B*	*TFAP2B*	*TFAP2B*	*PTGIS*
	rs760900	rs987237	rs2817399	rs2817416	rs2817419	rs2635727	Haplotype	760900	987237	2817399	2817416	2817419	2635727	Haplotype
	C	G	A	C	G	T		C	G	A	C	G	T	
**Ca**^**2+**^ **signaling**														
ATP2A3/SERCA	−0.032**	−0.031**	−0.029*	−0.033**	−0.031**	−0.029*	−0.032**							
CACNA1C/CaV1.2	−0.056**	−0.058**	−0.052**	−0.059**	−0.056**	−0.053**	−0.055**							
CACNA1G/Cav3.1	−0.136**	−0.139**	−0.138**	−0.141**	−0.137**	−0.137**	−0.139**	0.164						
CACNB2/Cavbeta2	−0.061**	−0.062**	−0.061**	−0.064**	−0.062**	−0.059**	−0.061**							
RHOB	0.039**	0.042**	0.042**	0.041**	0.041**	0.044**	0.041**							
ROCK1	0.048*	0.042	0.031	0.036	0.042	0.044*	0.043							
**K**^**+**^ **channels**														
KCNA2/Kv1.2	−0.208**	−0.203**	−0.204**	−0.207**	−0.207**	−0.195**	−0.201**							
KCNA5/Kv1.5														
KCNB1/Kv2.1	−0.076**	−0.081**	−0.075**	−0.086**	−0.081**	−0.075**	−0.08**							
KCNS3/Kv9.3	−0.235**	−0.236**	−0.246**	−0.241**	−0.233**	−0.222**	−0.239**							
KCNJ8/Kir6.1	0.034*	0.032*		0.028	0.032*	0.04**	0.036*							
ABCC9/SUR2B	0.056**	0.05**	0.043**	0.045**	0.051**	0.055**	0.051**							
**Contractile proteins**														
ACTA2	0.068**	0.069**	0.07**	0.066**	0.068**	0.071**	0.069**							
CNN1/Calponin	0.125**	0.121**	0.119**	0.121**	0.122**	0.124**	0.122**							
MYH11/SM1	0.101**	0.095**	0.092**	0.095**	0.098**	0.099**	0.095**							
MYH11/SM2	0.148**	0.146**	0.137**	0.144**	0.146**	0.148**	0.144**							
MYLK	0.063**	0.063**	0.062**	0.057**	0.063**	0.066**	0.063**							
MYOCD/Myocardin	−0.044**	−0.045**	−0.038**	−0.042**	−0.045**	−0.041**	−0.044**							
TPM1/Tropomyosin	0.044**	0.041**	0.045**	0.038**	0.042**	0.045**	0.042**							
**Endothelin signaling**														
ECE1	0.028**	0.025**	0.021**	0.022**	0.026**	0.027**	0.024**							
EDNRA/EtA-receptor	−0.066**	−0.068**	−0.068**	−0.073**	−0.067**	−0.067**	−0.068**							
EDNRB/EtB-receptor	−0.068**	−0.066**	−0.073**	−0.074**	−0.066**	−0.064**	−0.068**							
**Prostaglandin signaling**														
PTGS1/COX1	0.09**	0.084**	0.091**	0.084**	0.085**	0.087**	0.086**							
PTGS2/COX2	0.05**	0.048**	0.047**	0.041**	0.049**	0.053**	0.047**	0.287**	0.238*	0.24*	0.243*	0.278**	0.241*	0.25*
CYP8A1/PTGIS	0.045**	0.041**	0.04**	0.04**	0.041**	0.044**	0.042**							
PTGER4/EP4	0.074**	0.07**	0.067**	0.069**	0.069**	0.072**	0.07**							
PDE1B	−0.051**	−0.053**	−0.052**	−0.055**	−0.052**	−0.048**	−0.053**							
PDE3B	0.038**	0.039**	0.039**	0.039**	0.038**	0.043**	0.039**							
PDE4D	0.044**	0.037**	0.031*	0.031*	0.037**	0.039**	0.036**							
SLCO2A1/PG transporter	0.066**	0.055**	0.045**	0.052**	0.055**	0.055**	0.056**	−0.311**	−0.364**	−0.323**	−0.353**	−0.37**	−0.355**	−0.361**
**Nitric oxide signaling**														
NOS3/eNOS	0.068**	0.064**	0.06**	0.059**	0.065**	0.067**	0.065**							
PDE5A														
**Inflammation and remodeling**														
AGTR1	−0.134**	−0.139**	−0.141**	−0.142**	−0.139**	−0.132**	−0.136**							
AGTR2	0.107**	0.101**	0.09**	0.09**	0.099**	0.102**	0.101**							
BMP4	−0.047**	−0.049**	−0.042**	−0.053**	−0.048**	−0.044**	−0.048**							
BMP9	−0.345**	−0.349**	−0.331**	−0.338**	−0.333**	−0.322**	−0.321**							
BMP10	−0.312**	−0.304**	−0.302**	−0.296**	−0.295**	−0.286**	−0.282**							
EPAS1/HIF2 alpha	0.059**	0.059**	0.056**	0.056**	0.059**	0.059**	0.059**							
FN1	0.022**	0.021**	0.021**	0.02*	0.021**	0.022**	0.02**							
IGF1	−0.214**	−0.218**	−0.209**	−0.216**	−0.215**	−0.207**	−0.21**							
ILK	0.075**	0.068**	0.062**	0.059**	0.068**	0.072**	0.066**							
JAG1	−0.045**	−0.047**	−0.044**	−0.049**	−0.045**	−0.048**	−0.047**							
MAPK1/ERK2	0.023*	0.019	0.016*	0.016*	0.02	0.023*	0.019							
PDGFB/PDGF-B chain	−0.042**	−0.045**	−0.042**	−0.051**	−0.043**	−0.039**	−0.045**							
PTPN11		−*0.015*		−0.018*	−*0.014*									
SMARCA4/BRG1	−0.348**	−0.034**	−0.032**	−0.036**	−0.033**	−0.031**	−0.034**							
TGFB1/TGF beta1	0.051**	0.046**	0.051**	0.039**	0.048**	0.049**	0.047**							
TFAP2B/TFAP2 beta	0.049**	0.046**	0.031**	0.045**	0.045**	0.048**	0.046**							
TRAF1														

Regression coefficients for gestation and non-European genetic ancestry were derived in the multivariable models described in “Methods”. Each model included the SNP of interest, gestational age, and genetic ancestry.

^a^Positive regression coefficients represent the increase in a gene’s ΔCT for every increased week of gestation; negative regression coefficients represent the decrease in a gene’s ΔCT for every increased week of gestation.

^b^Regression coefficients represent the increase in a gene’s ΔCT when ductus from fetuses with non-European ancestry were compared to those from fetuses with European ancestry.

Regression coefficients are listed in the table if the association with the “ductus closure gene” has a *p* value < 0.10. Negative regression coefficients are in italics. *p < 0.05; **p < 0.01.

Total RNA was isolated from each individual DA and cDNA was generated as described elsewhere.^6,17^ We used the TaqMan Universal PCR master mix of PE Applied Biosystems (Foster City, CA) to quantify gene expression in a 96-well format. TaqMan probes were designed using the Primer Express program and labeled with fluorophores FAM (6-caboxy-fluorescein) and TAMRA (6 carboxy-tetramethyl-rhodamine) as reporter and quencher dyes, respectively. An ABI PRISM 7500 Sequence detection system was used to determine the cycle threshold (CT). Reactions were carried out in triplicate. Data were analyzed using the Sequence Detector version 1.6.3 program. The degree of expression of the gene of interest was determined using the relative gene expression method. Malate dehydrogenase (MDH) was used as an internal control to normalize the data.^6,18^ ΔCT represents the difference in cycle threshold (CT) between the expression of the housekeeping gene (MDH) and the gene of interest. Each unit of ΔCT represents a twofold change in mRNA levels. The more negative the ΔCT, the fewer the number of starting copies of a gene’s mRNA.

### DNA genotyping of fetal ductus arteriosus to determine the presence or absence of several TFAP2B and PTGIS SNPs as well as to infer genetic ancestry

DNA was extracted from the ascending aorta of each of the fetal samples using the QIAamp DNA mini kit (Qiagen Inc., Valencia, CA). DNA was quantified spectrophotometrically. Allelic variation was determined by using the TaqMan genotyping system (Applied Biosystems, Foster City, CA), as previously described.^19^ Allele scoring was performed using the Sequence Detection Systems 2.2 software (Applied Biosystems).

**Fig. 1:**
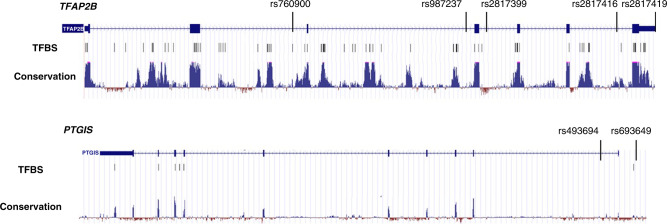
Gene structure of human TFAP2B and PTGIS and location of polymorphisms. The TFAP2B polymorphism rs2635727 is located further to the right of the gene and is not shown on the figure. In contrast to PTGIS, the intronic regions of TFAP2B show many areas of conservation, some with consensus transcription factor-binding sites. Figures were obtained from the UCSC Genome Browser. Note that the two genes are represented in opposite orientations and not to the same scale. The exons of each gene are shown as the thick lines at the top of the figure. TFBS = conserved transcription factor-binding sites; conservation estimated sequence = conservation of genetic loci among 100 vertebrate species (PhyloP).^24^

We examined the DNA for the presence of several SNPs in the *TFAP2B* and *PTGIS* genes that have been associated with altered DA closure in preterm infants (Fig. 1)^10^ (Dagle et al., unpublished results). Specifically, we examined the DNA for four SNPs in *TFAP2B* (rs2817399: (A allele); rs987237: (G allele); rs760900: (C allele); rs2817416 (C allele)) that have been associated with delayed DA closure (even in the presence of indomethacin). We also determined the presence of two *TFAP2B* SNPs that are unrelated to the timing of DA closure (rs2817419: G allele; and rs2635727: T allele). In addition, we examined one haplotype combination of two neighboring SNPs in the gene *PTGIS* (rs493694 (G allele) and *PTGIS* rs693649 (A allele)) that is negatively associated with PDA, suggesting a preventative effect of the allele combination against PDA.^10^

To identify the genetic ancestry of the fetal tissues, we examined four genes whose sequence polymorphisms are distributed along genetic lines between European ancestry and Non-European ancestry (African, Chinese, and Japanese) populations.^20–22^ These include *SLC24A5* SNP rs1426654 (100% European **=** **A** allele; 98% Non-European = **G** allele)^20,21^; *SLC45A2* SNP rs16891982 (100% European = **G** allele; 100% Non-European = **C** allele)^20,21^; *DARC* SNP rs2814778 (100% European = **A** allele; 85–100% Non-European = **G** allele)^22^; and *HERC2* SNP rs12913832 (79% European = **G** allele; 100% Non-European = **A** allele).^21^ Each fetal sample possessed between 0 and 8 of the Non-European ancestry alleles. Although we recognized that the clinic’s population data for race was an imperfect proxy for genetic ancestry, we used the clinic’s statistics to help create a definition for European and Non-European ancestry. Since 76% of the women who donated tissue self-identified as Non-White/Non-European ancestry and 83% of the samples had two or more Non-European ancestry alleles (see “Results”), we defined tissues with two or more Non-European ancestry alleles as “Non-European origin” and those with zero or one Non-European ancestry allele as “European origin” (The bold lettering is to highlight the only change in the SNPs).

### Statistical analyses

Stata software (Release 16.1; StataCorp LP, College Station, TX) was used for all statistical analyses. We used multivariable linear regression to build statistical models that adjusted for possible confounding effects of gestational age and genetic ancestry on the relationship between a SNP, or 2-SNP haplotype, and the change in RNA expression of each of the 49 “DA closure genes” (represented by their ΔCT). The multivariable models were analyzed using generalized estimating equations techniques to account for clustering within each of the 90 sample “batch” assays. Coefficients derived from these models were interpreted as the difference (positive or negative) between the RNA expression in the presence of the SNP in the study population and that in the absence of the SNP while holding gestational age and genetic ancestry constant. Models were run individually for each of the 49 “DA closure genes”.

To determine if an SNP’s effect on RNA expression differed depending on whether it occurred in a European ancestry or non-European ancestry DA, we added an interaction term to the model (between the SNP in question and genetic ancestry) and reran the regression.

Our study was an exploratory study designed to identify “DA closure genes” that might be altered by the presence of common genetic variants. Because of its exploratory nature, we considered any association between an SNP and a change in gene expression as possible evidence of association if the regression coefficient for RNA expression had a *p* value < 0.1. Our purpose was to decrease the likelihood of missing true positive signals, knowing that false-positive signals will inevitably be present.

## Results

We analyzed 273 fetal DA samples in the current study (gestational age = 19.8 ± 2.9 weeks (m ± s.d.)). Seventeen percent of the samples had zero or one non-European ancestry allele and were assigned as European ancestry. The allele frequencies of the *TFAP2B* polymorphisms associated with an increased incidence of PDA were as follows: rs2817399 (A allele) = 81%; rs987237 (G allele) = 37%; rs760900 (C allele) = 89%; and rs2817416 (C allele) = 25%). The frequencies of the *TFAP2B* polymorphisms that are unrelated to the timing of DA closure were: (rs2817419 (G allele) = 42% and rs2635727 (T allele) = 38%). The frequency of the *PTGIS* haplotype of two neighboring SNPs that is negatively associated with PDA was: (rs493694 (G allele)/rs693649 (A allele) = 21%).

We created multivariable linear regression models to determine the independent effects of gestational age, genetic ancestry, and the SNP alleles on RNA expression of the 49 “DA closure genes”. As previously reported, advancing gestational age was independently associated with changes in RNA expression for the majority (92%) of the “DA closure genes” (Table 1). In contrast, genetic ancestry was only consistently and independently associated with RNA expression in 2 genes: *PTGS2/COX2* (cyclooxygenase 2) and *SLOCA2A1* (the prostaglandin transporter which regulates prostaglandin reuptake) (Table 1).

Our main objective was to identify “DA closure genes” that are modified by the *TFAP2B* and *PTGIS* SNPs that have previously been shown to alter DA behavior: rs2817399 (A allele), rs987237 (G allele), rs760900 (C allele), and rs2817416 (C allele). In our initial examination of the general population of 273 samples, we found no consistent independent association between the *TFAP2B* SNPs associated with delayed DA closure and alterations in RNA expression for any of the “DA closure genes” (Table 2—General population).

**Table 2:** Multivariable regression models examining the independent effects of TFAP2B SNPs (associated with persistent PDA) on the RNA expression of “ductus closure genes” in second trimester human ductus (*n* = 273).

Genes/Aliases	Regression coefficients for *TFAP2B* (PDA-associated polymorphisms)												Regression coefficients for *TFAP2B* (non-PDA-associated polymorphisms)	
	General population^a^				European ancestry^b^				Non-European ancestry^c^				European ancestry^b^	
	rs760900	rs987237	rs2817399	rs2817416	rs760900	rs987237	rs2817399	rs2817416	rs760900	rs987237	rs2817399	rs2817416	rs2817419	rs2635727
	C	G	A	C	C	G	A	C	C	G	A	C	G	T
**Ca**^**2+**^ **signaling**														
ATP2A3/SERCA					−0.444*	−0.511**	−0.411*			0.126				
CACNB2/Cavbeta2	−0.215*				**−*0.357***	−0.493**	**−*0.361***							
**K**^**+**^ **channels**														
KCNA2/Kv1.2					−1.361*	−1.301**	**−*1.084***							
KCNS3/Kv9.3													1.361	1.342
KCNJ8/Kir6.1				−0.353**				−0.267				−0.385**		
ABCC9/SUR2B				−0.194*								−0.212*		
**Contractile proteins**														
CNN1/Calponin	0.235*				0.364*	0.25								
MYH11/SM1					0.832*		0.773**						0.649*	0.712*
MYH11/SM2					0.509*		0.381**							
**Endothelin signaling**														
ECE1					−0.281**	−0.174*	−0.243*	−0.272**					0.209*	0.191
EDNRA/EtA-receptor	−0.258**			−*0.109*	**−*0.329***	**−*0.228***	**−*0.293***	**−*0.27***	−0.231*					
EDNRB/EtB-receptor	−*0.237*			−0.206*	**−*0.394***	−0.515*		−0.58*					0.339	
**Prostaglandin Signaling**														
PTGS1/COX1					0.207		0.404							
PTGS2/COX2						−0.422**							0.348	0.341
PDE1B						−0.328*								
PDE3B						−*0.35*								
SLCO2A1/PG transporter	−0.259*				−*0.361*				−*0.221*					
**Nitric oxide signaling**														
NOS3/eNOS						−*0.297*								
**Inflammation and remodeling**														
AGTR1						−0.765*				0.37*	0.379*			
BMP4						−0.361*								
BMP9	−1.13*				−2.079**	−1.841*	−1.909*							
BMP10	−0.891*				−1.531**	−1.598**	−1.438*							
EPAS1/HIF2 alpha					**−*0.238***		**−*0.235***	−0.341**						
IGF1						−0.937*	−0.92			0.338				
PDGFB/PDGF-B chain						−0.389*								
PTPN11						−*0.208*				0.111				
SMARCA4/BRG1						−*0.173*		−*0.198*					0.172	
TRAF1						−*0.346*								

The bold values indicate the genes where at least three of the four TFAP2B SNPs were associated with changes in expression.

Regression coefficient represents the increase in a gene’s ΔCT when the TFAP2B allele was present (compared with when it was absent). Regression coefficients were calculated for each of the 49 “ductus closure genes” listed in Table 1. Regression coefficients are only listed in the table if the association with the “ductus closure gene” has a *p* value < 0.10. Negative regression coefficients are in italics. *p < 0.05; **p < 0.01.

^a^General population: multivariate analyses were adjusted for gestational age and genetic ancestry (European or Non-European) without an interaction term between the SNP allele and genetic ancestry.

^b^European ancestry: multivariate analyses were adjusted for gestational age and genetic ancestry (European or Non-European) plus an interaction term between the SNP allele and genetic ancestry; regression coefficients were obtained for the referent value: European genetic ancestry.

^c^Non-European ancestry: multivariate analyses were adjusted for gestational age and genetic ancestry (European or Non-European) plus an interaction term between the SNP allele and genetic ancestry; regression coefficients were obtained for the referent value: non-European genetic ancestry.

However, when we tested whether an interaction occurred between the fetus’s genetic ancestry and the same PDA-associated *TFAP2B* SNPs, we found that several of the “DA closure genes” had consistent, independent changes in gene expression when the SNPs occurred in samples with European ancestry. At least three of the four *TFAP2B* SNPs were associated with changes in expression in each of the following genes: *EPAS1* (HIF2 alpha), *CACNB2* (Cavbeta2 calcium channel subunit), *ECE1* (endothelin converting enzyme), *KCNA2* (potassium channel Kv1.2), *ATP2A3* (SERCA, sarcoplasmic reticulum Calcium-ATPase), *EDNRA* (endothelin A-receptor), *EDNRB* (endothelin B-receptor), *BMP9* (bone morphogenetic protein-9), and *BMP10* (bone morphogenetic protein-10) (Table 2—European ancestry). None of these changes were seen when the same SNPs were examined in the samples with Non-European ancestry (Table 2—Non-European ancestry). Nor were the same changes observed when the two *TFAP2B* polymorphisms that are unrelated to the timing of DA closure (rs2817419 (G allele) and rs2635727 (T allele)) were examined in samples with European ancestry (Table 2—European ancestry/*TFAP2B (*Non-PDA-associated polymorphisms)).

A similar phenomenon occurred when we tested whether an interaction occurred between the fetus’s genetic ancestry and the 2-SNP haplotype of *PTGIS* that is negatively associated with PDA (rs493694 (G allele)/rs693649 (A allele)). When the *PTGIS* haplotype was present in samples with European ancestry, the haplotype was associated with changes in RNA expression in several “DA closure genes” (the most significant change occurring in *PTGIS* itself) (Table 3).

**Table 3:** Multivariable regression models examining the independent effects of the *PTGIS* SNP haplotype rs493694(G)/rs693649(A) (associated with early ductus closure) on the RNA expression of “ductus closure genes” (*n* = 273).

Genes/aliases	Regression coefficients for PTGIS	
	Haplotype: rs493694(G)/rs693649(A)	
	General population^a^	European ancestry^b^
Ca^2+^ signaling		
CACNB2/Cavbeta2		−0.469*
K^+^ channels		
KCNS3/Kv9.3		−2.454*
Contractile proteins		
MYH11/SM1		*−0.636*
Endothelin signaling		
Prostaglandin signaling		
CYP8A1/PTGIS		−0.461**
Nitric oxide signaling		
NOS3/eNOS		−0.618*
Inflammation and remodeling		
BMP9		−2.22*
BMP10	*−0.513*	−1.36*

Regression coefficient represents the increase in a gene’s ΔCT when the *PTGIS* SNP haplotype was present (compared with when it was absent). Regression coefficients were calculated for each of the 49 “ductus closure genes” listed in Table 1. Regression coefficients are only listed in the table if the association with the “ductus closure gene” has a *p* value < 0.10. Negative regression coefficients are in italics. *p < 0.05; **p < 0.01.

^a^General population: multivariate analyses were adjusted for gestational age and genetic ancestry (European or Non-European) without an interaction term between the haplotype and genetic ancestry.

^b^European ancestry: multivariate analyses were adjusted for gestational age and genetic ancestry (European or Non-European) plus an interaction term between the haplotype and genetic ancestry; regression coefficients were obtained for the referent value: European genetic ancestry.

## Discussion

Premature infants born to mothers who self-identify as White/European ancestry are less likely to close their PDA following prostaglandin inhibition than infants born to mothers who self-identify as Non-White/Non-European ancestry.^1–4^ This difference does not appear to be due to different rates of indomethacin/ibuprofen metabolism or different serum prostaglandin E2 concentrations.^1–4^ Our current study demonstrates that genetic ancestry is associated with changes in the expression of several “DA closure genes”. This occurs through a direct association between genetic ancestry and a limited number of “DA closure genes” (*SLCO2A1* (the prostaglandin transporter) and *PTGS2* (cyclooxygenase 2)) (Table 1), as well as through a broader, indirect, interactive effect, where genetic ancestry modifies the associations between common genetic polymorphisms and DA gene expression.

We previously identified several polymorphisms in the genes *PTGIS* and *TFAP2B* that were associated with different rates of PDA closure in a population composed primarily of preterm infants with European genetic ancestry.^10^ These associations were not replicated by other investigators using populations with different or more diverse genetic origins.^14,15^ In line with these discordant observations, our current study found consistent associations between *PTGIS* and *TFAP2B* polymorphisms and the expression of “DA closure genes” in DA with European genetic ancestry. On the other hand, no consistent positive or negative associations could be found in our genetically diverse DA population unless an interaction between the polymorphisms and genetic ancestry was taken into account (Tables 2 and 3).

In DA with European genetic ancestry, the *PTGIS* haplotype (rs493694 (G allele)/rs693649 (A allele)), which is associated with early DA closure, was associated with decreased expression of *PTGIS* itself as well as *NOS3* (endothelial nitric oxide synthase, which regulates nitric oxide production) and several other calcium and potassium regulatory genes (Table 3).

Consistent alterations in gene expression were also found when each of the four *TFAP2B* SNPs (that are associated with persistent PDA) were present in DA with European genetic ancestry. These changes include decreased expression of calcium and potassium signaling genes, as well as decreased expression of genes regulating endothelin and HIF2 alpha (Table 2). It is interesting to note that similar changes in endothelin and HIF2 alpha were previously found in newborn mice with targeted deletions of Tfap2b (the mouse equivalent of *TFAP2B).*^12^

To determine whether the changes in DA gene expression were specific for the *TFAP2B* SNPs associated with persistent PDA, we examined two other *TFAP2B* polymorphisms, rs2817419(G) and rs2635727(T), which are unrelated to the incidence of preterm PDA (Table 2). Neither polymorphism was associated with the changes in gene expression described above (Table 2).

Our study has several limitations. The tissues were from pregnancy terminations, which may have altered the gene expression in the DA before tissue processing. We explored a limited number of candidate genes and may have missed others that might have been detected by genome-wide association studies or pathway-based analyses. There was also a relatively small number of tissue samples and a low proportion of European genetic ancestry in our study population which may have limited our ability to identify smaller effects in the “DA closure genes” we studied.

Since our investigation was an exploratory study, we chose to consider results with a *p* value < 0.1 as possible evidence of association. Although applying a more stringent *p* value would have reduced the chance of finding false-positive signals, it might have eliminated our ability to detect true positive signals, especially when the genetic effects are small. Our finding that at least three of the four *TFAP2B* SNPs, that were associated with persistent PDA, also were associated with the same changes in expression of several of the “DA closure genes” (*EPAS1*, *CACNB2*, *ECE1*, *KCNA2*, *ATP2A3*, *EDNRA*, *EDNRB*, *BMP9*, and *BMP10*) increases the confidence that these may actually represent true positive results. None of these changes were seen when the two *TFAP2B* polymorphisms that were unrelated to the timing of DA closure were examined in samples with European genetic ancestry (Table 2).

As an observational study, we cannot distinguish between causation and association. Nor do we know if the changes in gene expression have a direct effect on DA closure, or if they are merely an indirect effect of other events that are responsible for its closure. However, our findings do provide biologic plausibility to the concept that the *PTGIS* and *TFAP2B* SNPs are either functional polymorphisms or in tight association with functional polymorphisms that play an active role in regulating DA closure. Since the SNPs we studied are present in haplotype blocks, the actual genetic variations responsible for the associated changes in gene expression could lie anywhere within that block. We speculate that the increased rate of DA closure associated with the *PTGIS* 2-SNP haplotype rs493694(G)/rs693649(A) may be due to the associated decrease in prostaglandin I2 synthase expression (and a subsequent decrease in the potent vasodilator, PGI2). On the other hand, we have no similar explanation for the changes associated with the *TFAP2B* SNPs since none of the SNPs appear to alter *TFAP2B* mRNA levels (Table 2). It is worth noting that the *TFAP2B* SNPs we examined are situated in unique, highly conserved regions, that are located between exons, and in proximity to a number of putative transcription factor-binding sites (Fig. 1). SNPs in or near a gene can affect both the amount and function of the mRNA or protein produced. We speculate that alterations in these unique, highly conserved, noncoding regions might alter TFAP2B splicing such that transcript levels are normal but the transcripts themselves are abnormal; or, they may have distant effects (possibly through altered transcription factor binding or microRNA production) on gene expression even beyond the *TFAP2B* gene in which they are located. These findings are consistent with our current understanding that many disease-associated common variants are noncoding and are enriched in DNA regulatory elements.^23^ Future studies will be needed to determine how these polymorphisms affect the expression of downstream genes.

In conclusion, we found no consistent associations between the presence of polymorphisms in *PTGIS* and *TFAP2B* and the expression of “DA closure genes” unless an interaction between the polymorphisms and genetic ancestry was taken into account. When an interaction between the polymorphisms and ancestry was accounted for, the *PTGIS* and *TFAP2B* polymorphisms were associated with consistent changes in DA gene expression in DA from fetuses with European genetic ancestry.

## Data Availability

The datasets generated and/or analyzed during the current study are available from the corresponding author on reasonable request.

## References

[1] Chorne N, Jegatheesan P, Lin E, Shi R, Clyman RI (2007). Risk factors for persistent ductus arteriosus patency during indomethacin treatment. J. Pediatr..

[2] Durrmeyer X (2010). Are cytochrome P450 CYP2C8 and CYP2C9 polymorphisms associated with ibuprofen response in very preterm infants?. PLoS ONE.

[3] Furzan JA, Reisch J, Tyson JE, Laird P, Rosenfeld CR (1985). Incidence and risk factors for symptomatic patent ductus arteriosus among inborn very-low-birth-weight infants. Early Hum. Dev..

[4] Cotton RB, Haywood JL, FitzGerald GA (1991). Symptomatic patent ductus arteriosus following prophylactic indomethacin. A clinical and biochemical appraisal. Biol. Neonate.

[5] Shelton EL (2018). Effects of antenatal betamethasone on preterm human and mouse ductus arteriosus: comparison with baboon data. Pediatr. Res..

[6] Waleh N (2010). Patterns of gene expression in the ductus arteriosus are related to environmental and genetic risk factors for persistent ductus patency. Pediatr. Res.

[7] Waleh N (2015). Effects of advancing gestation and non-caucasian race on ductus arteriosus gene expression. J. Pediatr..

[8] Bhandari V (2009). Genetic contribution to patent ductus arteriosus in the premature newborn. Pediatrics.

[9] Lavoie PM, Pham C, Jang KL (2008). Heritability of bronchopulmonary dysplasia, defined according to the consensus statement of the National Institutes of Health. Pediatrics.

[10] Dagle JM (2009). Determination of genetic predisposition to patent ductus arteriosus in preterm infants. Pediatrics.

[11] Majed BH, Khalil RA (2012). Molecular mechanisms regulating the vascular prostacyclin pathways and their adaptation during pregnancy and in the newborn. Pharmacol. Rev..

[12] Ivey KN (2008). Transcriptional regulation during development of the ductus arteriosus. Circ. Res..

[13] Zhao F (2001). Novel TFAP2B mutations that cause Char syndrome provide a genotype-phenotype correlation. Am. J. Hum. Genet..

[14] Kawase K (2016). Single nucleotide polymorphisms in AGTR1, TFAP2B, and TRAF1 are not associated with the incidence of patent ductus arteriosus in Japanese preterm infants. Pediatr. Int.

[15] Dagle JM (2019). Genetic variants associated with patent ductus arteriosus in extremely preterm infants. J. Perinatol..

[16] Merz E, Oberstein A, Wellek S (2000). Age-related reference ranges for fetal foot length. Ultraschall Med..

[17] Bouayad A (2001). Characterization of PGE2 receptors in fetal and newborn lamb ductus arteriosus. Am. J. Physiol. Heart Circ. Physiol..

[18] Waleh N (2004). Prostaglandin E2-mediated relaxation of the ductus arteriosus: effects of gestational age on g protein-coupled receptor expression, signaling, and vasomotor control. Circulation.

[19] Ehn NL (2007). Evaluation of fetal and maternal genetic variation in the progesterone receptor gene for contributions to preterm birth. Pediatr. Res..

[20] Soejima M, Koda Y (2007). Population differences of two coding SNPs in pigmentation-related genes SLC24A5 and SLC45A2. Int J. Leg. Med..

[21] Beleza S (2013). Genetic architecture of skin and eye color in an African-European admixed population. PLoS Genet.

[22] Reiner AP (2005). Population structure, admixture, and aging-related phenotypes in African American adults: the Cardiovascular Health Study. Am. J. Hum. Genet..

[23] Maurano MT (2012). Systematic localization of common disease-associated variation in regulatory DNA. Science.

[24] Siepel A (2005). Evolutionarily conserved elements in vertebrate, insect, worm, and yeast genomes. Genome Res..

